# Supplementary figures and images for: Exploring the changing association between parental and adolescent fruit and vegetable intakes, from age 10 to 30 years
Source: Int J Behav Nutr Phys Act. 2024 May 10;21:56. doi: 10.1186/s12966-024-01604-8 (PMC11083755; doi:10.1186/s12966-024-01604-8)

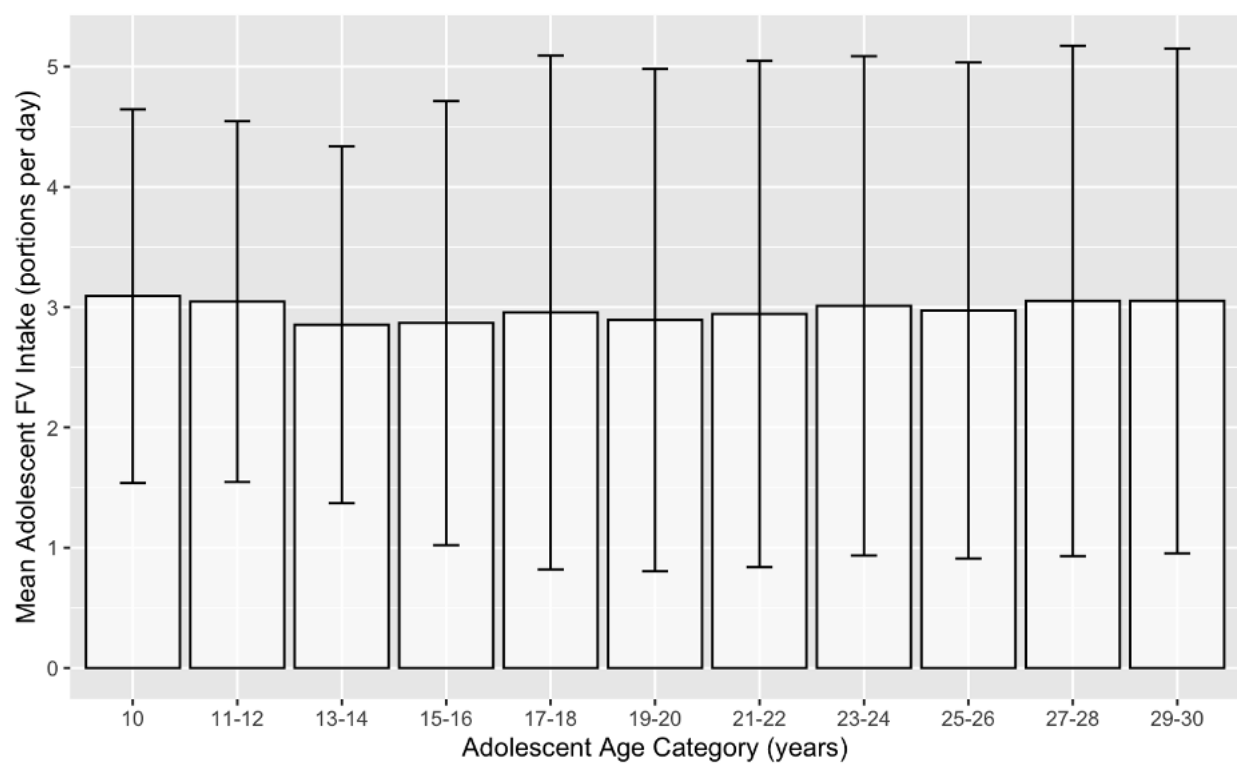

Supplement: Supplementary file 1 — Supplementary Material 1. Mean adolescent FV intake (portions per day) by age category (n = 12,805). Error bars represent ± the standard deviation. [file 12966_2024_1604_MOESM1_ESM.pdf]

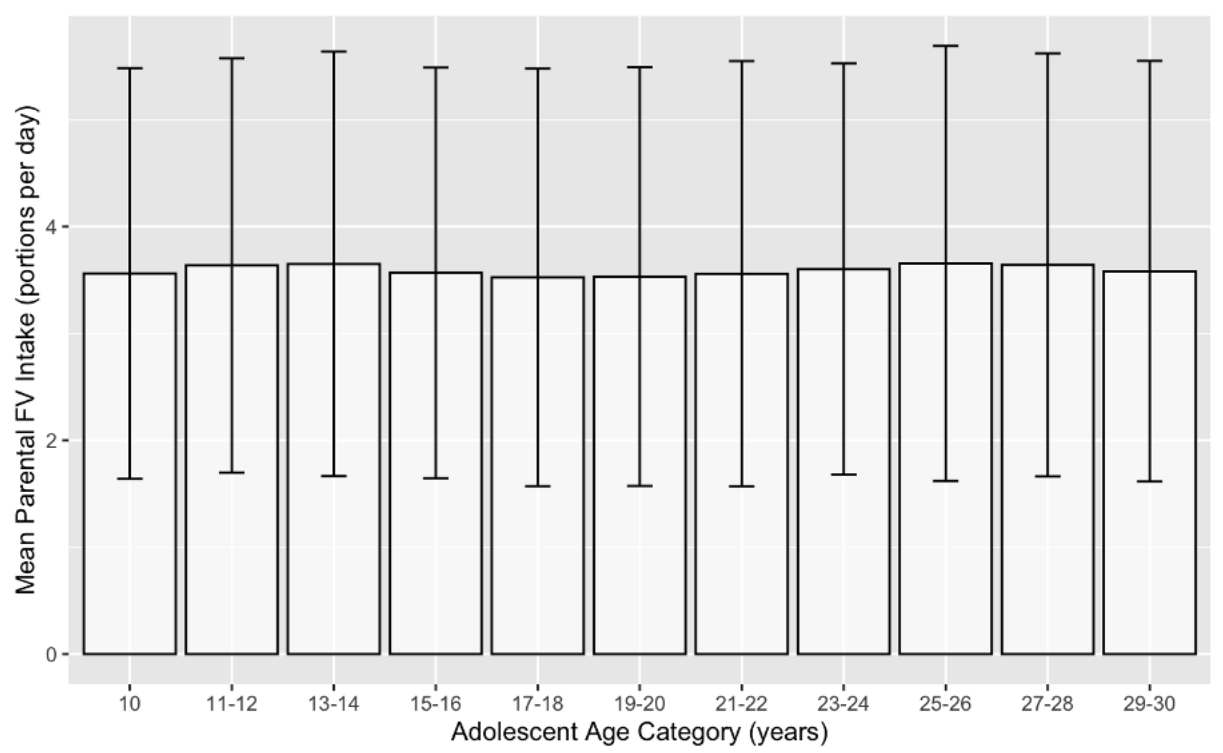

Supplement: Supplementary file 2 — Supplementary Material 2. Mean parental FV intake (portions per day) by adolescent age category (n = 12,805). Error bars represent ± the standard deviation. [file 12966_2024_1604_MOESM2_ESM.pdf]
