# Supplementary material for: Exploring the changing association between parental and adolescent fruit and vegetable intakes, from age 10 to 30 years
Source: Int J Behav Nutr Phys Act. 2024 May 10;21:56. doi: 10.1186/s12966-024-01604-8 (PMC11083755; doi:10.1186/s12966-024-01604-8)
Supplement: Supplementary file 3 — Supplementary Material 3. [file 12966_2024_1604_MOESM3_ESM.pdf]

	Unadjusted	Adjusted for covariates	Adjusted for age categories	Interactions with age categories	Interactions with living arrangement	Stratified by living arrangement	
	**Model 1**	**Model 2**	**Model 3**	**Model 4**	**Model 5**	**Model 5a (living in the parental home)**	**Model 5b (not living in the parental home)**
**Parameters**				Estimates [95% CI]			
(Intercept)	2.14 *** [2.09, 2.19]	2.47 *** [2.36, 2.58]	2.87 *** [2.70, 3.04]	3.18 *** [2.95, 3.42]	3.18 *** [2.95, 3.41]	2.12 *** [1.92, 2.32]	2.78 *** [1.61, 3.95]
Parental FV Intake (portions per day)	0.23 *** [0.21, 0.24]	0.20 *** [0.19, 0.22]	0.20 *** [0.19, 0.22]	0.12 *** [0.07, 0.16]	0.12 *** [0.07, 0.16]	0.30 *** [0.26, 0.33]	0.20 [-0.07, 0.48]
**Covariates**							
Sex – Female		0.27 *** [0.22, 0.33]	0.27 *** [0.22, 0.33]	0.27 *** [0.22, 0.33]	0.27 *** [0.21, 0.32]	0.34 *** [0.26, 0.43]	0.24 ** [0.08, 0.40]
Ethnicity – Non-white		-0.27 *** [-0.35, -0.20]	-0.27 *** [-0.35, -0.20]	-0.28 *** [-0.35, -0.21]	-0.26 *** [-0.33, -0.19]	-0.36 *** [-0.47, -0.26]	-0.43 ** [-0.69, -0.17]
Parental education – No degree		-0.43 *** [-0.49, -0.38]	-0.43 *** [-0.48, -0.37]	-0.43 *** [-0.49, -0.37]	-0.43 *** [-0.48, -0.37]	-0.52 *** [-0.60, -0.43]	-0.50 *** [-0.65, -0.34]
Household income (£GBP per month)		0.00 *** [0.00, 0.00]	0.00 *** [0.00, 0.00]	0.00 *** [0.00, 0.00]	0.00 *** [0.00, 0.00]	0.00 ** [0.00, 0.00]	0.00 * [0.00, 0.00]
Geographic Region (ref: London)							
North East		-0.34 *** [-0.51, -0.17]	-0.34 *** [-0.51, -0.17]	-0.35 *** [-0.52, -0.18]	-0.35 *** [-0.51, -0.18]	-0.34 * [-0.60, -0.07]	-0.77 *** [-1.21, -0.33]
North West		-0.23 *** [-0.34, -0.12]	-0.23 *** [-0.34, -0.12]	-0.23 *** [-0.34, -0.12]	-0.23 *** [-0.35, -0.12]	-0.24 ** [-0.41, -0.06]	-0.58 *** [-0.91, -0.25]
Yorkshire and the Humber		-0.26 *** [-0.38, -0.15]	-0.27 *** [-0.38, -0.15]	-0.26 *** [-0.38, -0.15]	-0.27 *** [-0.38, -0.15]	-0.29 ** [-0.47, -0.11]	-0.53 ** [-0.85, -0.20]
East Midlands		-0.12 [-0.25, 0.01]	-0.12 [-0.25, 0.01]	-0.12 [-0.25, 0.01]	-0.12 [-0.25, 0.00]	-0.09 [-0.29, 0.10]	-0.46 ** [-0.80, -0.11]
West Midlands		-0.10 [-0.21, 0.02]	-0.10 [-0.21, 0.01]	-0.09 [-0.20, 0.02]	-0.09 [-0.20, 0.02]	-0.07 [-0.24, 0.10]	-0.40 * [-0.75, -0.05]
East of England		-0.08 [-0.20, 0.05]	-0.08 [-0.20, 0.04]	-0.07 [-0.20, 0.05]	-0.08 [-0.20, 0.04]	0.03 [-0.16, 0.22]	-0.49 ** [-0.85, -0.14]
South East		0.01 [-0.11, 0.12]	0.00 [-0.11, 0.11]	0.01 [-0.10, 0.12]	0.01 [-0.10, 0.12]	0.04 [-0.13, 0.22]	-0.42 ** [-0.73, -0.11]
South West		0.01	0.01	0.02	0.02	0.01	-0.43 *

	[-0.11, 0.14]	[-0.12, 0.14]	[-0.11, 0.15]	[-0.11, 0.15]	[-0.20, 0.22]	[-0.76, -0.09]
Wales	-0.22 **	-0.22 **	-0.22 **	-0.22 **	-0.14	-0.50 **
	[-0.36, -0.08]	[-0.36, -0.08]	[-0.36, -0.08]	[-0.36, -0.08]	[-0.36, 0.07]	[-0.86, -0.14]
Scotland	-0.16 *	-0.16 *	-0.16 *	-0.16 *	-0.24 *	-0.39 *
	[-0.29, -0.03]	[-0.29, -0.03]	[-0.29, -0.03]	[-0.29, -0.03]	[-0.44, -0.03]	[-0.72, -0.05]
Northern Ireland	-0.25 ***	-0.25 ***	-0.24 ***	-0.24 ***	-0.29 **	-0.74 ***
	[-0.38, -0.11]	[-0.38, -0.11]	[-0.38, -0.11]	[-0.37, -0.10]	[-0.49, -0.09]	[-1.14, -0.34]
**Age category (ref: 10 years)**						
11-12 years		-0.04	-0.05	-0.05		
		[-0.14, 0.05]	[-0.26, 0.16]	[-0.26, 0.16]		
13-14 years		-0.24 ***	-0.17	-0.17		
		[-0.34, -0.14]	[-0.39, 0.04]	[-0.39, 0.04]		
15-16 year		-0.36 ***	-0.62 ***	-0.62 ***		
		[-0.47, -0.24]	[-0.85, -0.40]	[-0.85, -0.40]		
17-18 years		-0.42 ***	-1.06 ***	-1.07 ***		
		[-0.57, -0.27]	[-1.30, -0.82]	[-1.31, -0.83]	**Ref: 17-18 years**	
19-20 years		-0.48 ***	-0.93 ***	-0.95 ***	0.10	0.11
		[-0.63, -0.33]	[-1.17, -0.68]	[-1.20, -0.71]	[-0.08, 0.28]	[-1.12, 1.34]
21-22 years		-0.43 ***	-0.95 ***	-1.02 ***	-0.02	0.44
		[-0.58, -0.29]	[-1.20, -0.71]	[-1.27, -0.78]	[-0.21, 0.17]	[-0.73, 1.61]
23-24 years		-0.37 ***	-0.82 ***	-0.94 ***	0.11	0.38
		[-0.52, -0.22]	[-1.07, -0.57]	[-1.20, -0.69]	[-0.10, 0.33]	[-0.78, 1.54]
25-26 years		-0.43 ***	-0.80 ***	-0.97 ***	0.10	0.29
		[-0.58, -0.27]	[-1.06, -0.55]	[-1.23, -0.71]	[-0.13, 0.34]	[-0.87, 1.45]
27-28 years		-0.39 ***	-0.78 ***	-1.01 ***	-0.01	0.32
		[-0.55, -0.23]	[-1.05, -0.51]	[-1.29, -0.74]	[-0.30, 0.28]	[-0.84, 1.48]
29-30 years		-0.36 ***	-0.78 ***	-1.03 ***	0.24	0.14
		[-0.53, -0.20]	[-1.06, -0.50]	[-1.32, -0.74]	[-0.09, 0.56]	[-1.03, 1.30]
**Interactions (ref: 10 years)**						
Parental FV X 11-12 years			0.00	0.00		
			[-0.05, 0.06]	[-0.05, 0.06]		
Parental FV X 13-14 years			-0.02	-0.02		
			[-0.07, 0.04]	[-0.07, 0.04]		
Parental FV X 15-16 years			0.07 **	0.07 **		
			[0.02, 0.13]	[0.02, 0.13]		
Parental FV X 17-18 years			0.18 ***	0.18 ***		
			[0.13, 0.23]	[0.13, 0.23]	**Ref: 17-18 years**	

Parental FV X 19-20 years				0.13 ***	0.13 ***	-0.05 *	0.00
				[0.07, 0.18]	[0.08, 0.19]	[-0.09, -0.00]	[-0.29, 0.29]
Parental FV X 21-22 years				0.15 ***	0.16 ***	-0.01	-0.06
				[0.09, 0.20]	[0.11, 0.22]	[-0.06, 0.04]	[-0.34, 0.22]
Parental FV X 23-24 years				0.13 ***	0.15 ***	-0.03	-0.05
				[0.07, 0.18]	[0.10, 0.21]	[-0.08, 0.03]	[-0.32, 0.23]
Parental FV X 25-26 years				0.10 ***	0.14 ***	-0.03	-0.05
				[0.05, 0.16]	[0.08, 0.20]	[-0.09, 0.02]	[-0.33, 0.23]
Parental FV X 27-28 years				0.11 ***	0.16 ***	0.00	-0.05
				[0.05, 0.17]	[0.10, 0.22]	[-0.07, 0.07]	[-0.33, 0.22]
Parental FV X 29-30 years				0.12 ***	0.17 ***	-0.06	-0.00
				[0.05, 0.18]	[0.11, 0.24]	[-0.14, 0.03]	[-0.28, 0.27]
Not living with parents					0.46 ***		
					[0.31, 0.61]		
Parental FV X Not living in the parental home					-0.10 ***		
					[-0.13, -0.06]		
N	26687	26687	26687	26687	26687	12158	3958
N (pidp)	12805	12805	12805	12805	12805	6979	2297
AIC	104366.28	103870.08	103819.32	103695.90	103662.60	49270.47	16066.14
BIC	104399.05	104033.92	104065.08	104023.57	104006.66	49500.05	16260.93
R2 (fixed)	0.05	0.09	0.09	0.09	0.09	0.12	0.07
R2 (total)	0.49	0.50	0.50	0.50	0.50	0.53	0.60

*** $p < 0.001$; ** $p < 0.01$; * $p < 0.05$.

Table S1: Multilevel linear regression model estimates. 95% significance level.
